# Antimicrobial and Conductive Nanocellulose-Based Films for Active and Intelligent Food Packaging

**DOI:** 10.3390/nano9070980

**Published:** 2019-07-06

**Authors:** Carla Vilela, Catarina Moreirinha, Eddy M. Domingues, Filipe M. L. Figueiredo, Adelaide Almeida, Carmen S. R. Freire

**Affiliations:** 1 Department of Chemistry, CICECO—Aveiro Institute of Materials, University of Aveiro, 3810-193 Aveiro, Portugal; 2 Department of Materials and Ceramic Engineering, CICECO—Aveiro Institute of Materials, University of Aveiro, 3810-193 Aveiro, Portugal; 3 Department of Biology and CESAM, University of Aveiro, 3810-193 Aveiro, Portugal

**Keywords:** bacterial nanocellulose, poly(sulfobetaine methacrylate), nanocomposite films, antimicrobial activity, moisture scavengers, active food packaging, protonic conductivity, intelligent food packaging

## Abstract

Bacterial nanocellulose (BNC) is becoming an important substrate for engineering multifunctional nanomaterials with singular and tunable properties for application in several domains. Here, antimicrobial conductive nanocomposites composed of poly(sulfobetaine methacrylate) (PSBMA) and BNC were fabricated as freestanding films for application in food packaging. The nanocomposite films were prepared through the one-pot polymerization of sulfobetaine methacrylate (SBMA) inside the BNC nanofibrous network and in the presence of poly(ethylene glycol) diacrylate as cross-linking agent. The ensuing films are macroscopically homogeneous, more transparent than pristine BNC, and present thermal stability up to 265 °C in a nitrogen atmosphere. Furthermore, the films have good mechanical performance (Young’s modulus ≥ 3.1 GPa), high water-uptake capacity (450–559%) and UV-blocking properties. The zwitterion film with 62 wt.% cross-linked PSBMA showed bactericidal activity against *Staphylococcus aureus* (4.3–log CFU mL^−1^ reduction) and *Escherichia coli* (1.1–log CFU mL^−1^ reduction), and proton conductivity ranging between 1.5 × 10^−4^ mS cm^−1^ (40 °C, 60% relative humidity (RH)) and 1.5 mS cm^−1^ (94 °C, 98% RH). Considering the current set of properties, PSBMA/BNC nanocomposites disclose potential as films for active food packaging, due to their UV-barrier properties, moisture scavenging ability, and antimicrobial activity towards pathogenic microorganisms responsible for food spoilage and foodborne illness; and also for intelligent food packaging, due to the proton motion relevant for protonic-conduction humidity sensors that monitor food humidity levels.

## 1. Introduction

The pervasive cellulose biopolymer is one of the most studied natural materials due to its renewability, unique set of properties and potential use in the most varied fields of application [1]. In fact, the domains of research are even broader when considering the nanoscale forms of cellulose, such as cellulose nanofibrils (CNFs), cellulose nanocrystals (CNCs) and bacterial nanocellulose (BNC) [2], with applications in catalysis [3], printed electronics [4], supercapacitors [5,6], sensing and biosensing [7], photonics, films, foams, nanocomposites and medical devices [8,9,10], and packaging materials [11,12], among many other examples [2,13].

BNC, a nanocellulose biosynthesized in nature [14], is an exopolysaccharide with the ability to lodge all kinds of molecules and macromolecules within its ultrafine nanofibrous network with the goal of preparing materials with distinct features and thus different application fields [15,16,17]. Therefore, the design of BNC nanocomposites [18] with smart and active functions is quite pertinent in the areas of active and intelligent food packaging [19,20,21]. While the former comprises packages with additives (e.g., antioxidant and antimicrobial agents, and moisture scavengers) that preserve or extend product quality or shelf-life [22], the latter encompasses indicators, data carriers and sensors that monitor the state of packaged food products [20]. As an illustrative example, Padrão et al. [23] fabricated BNC-lactoferrin edible films with high antimicrobial activity against *Staphylococcus aureus* for active food packaging, while Kuswandi et al. [24] produced an intelligent food package by combining BNC with methyl red that worked as a gas sensor for volatile amines produced steadily in the package headspace of broiler chicken cut.

Zwitterionic polymers with oppositely charged cationic and anionic groups are a singular class of adaptive materials with dual-nature properties, i.e., anti-polyelectrolyte or polyelectrolyte behavior, depending on the environmental stimuli, as recently reviewed by Blackman et al. [25]. Poly(sulfobetaine methacrylate) (PSBMA) is a widely known zwitterionic polymer consisting of a trimethylammonium cation and a sulfonate anion. Although PSBMA is mainly applied in biomedical applications, e.g., wound dressing [26,27] and skin regeneration [28], its antimicrobial properties against several microorganisms (e.g., *Pseudomonas aeruginosa* and *Staphylococcus epidermidis* [26], *S. aureus* and *Escherichia coli* [27]) and polyzwitterionic nature [29] could be a major advantage for the design of active and intelligent food packaging materials. However, to the best of our knowledge, the use of BNC in tandem with a zwitterionic polymer has not yet been investigated for engineering nanocomposite films with bioactivity and smart features, and thus with the ability for application in active and intelligent food packaging.

In view of our interest in bio-based materials for active food packaging [19,30,31], this study reports an endeavor to fabricate nanocellulose-based films with UV-light protection, antimicrobial activity, moisture control and proton motion for active and intelligent food packaging. The films were fabricated via the one-pot polymerization of sulfobetaine methacrylate (SBMA) inside the BNC nanofibrous network with poly(ethylene glycol) diacrylate as cross-linking agent, and characterized in terms of structure, morphology, thermal stability, mechanical performance, optical properties, antimicrobial activity against *S. aureus* and *E. coli*, moisture- and water-uptake capacity, and protonic conductivity.

## 2. Materials and Methods

### 2.1. Chemicals, Materials and Microorganisms

Sulfobetaine methacrylate (SBMA, 95%), potassium persulfate (KPS, ≥99%), poly(ethylene glycol) diacrylate (PEGDA, Mn 250), potassium sulfate (≥99.0%), tryptic soy broth (TSB), phosphate buffered saline (PBS, pH 7.4) and tryptic soy agar (TSA) were acquired from Sigma-Aldrich (Sintra, Portugal). Ultra-purified water (Type 1, 18.2 MΩ·cm resistivity (25 °C) at 0.5 L min^−1^) was provided by a Simplicity Water Purification System (Merck, Darmstadt, Germany).

Wet pellicles of bacterial nanocellulose (BNC) were biotechnologically produced in our research laboratory using the *Gluconacetobacter sacchari* bacterial strain [32]. *Staphylococcus aureus* (ATCC 6538) and *Escherichia coli* (ATCC 25922) were purchased from DSMZ—Deutsche Sammlung von Mikroorganismen und Zellkulturen GmbH (German Collection of Microorganisms and Cell Cultures, Braunschweig, Germany).

### 2.2. Fabrication of PSBMA/BNC Nanocomposite Films

Aqueous solutions of monomer (SBMA, 1:3 and 1:5 of BNC/SBMA weight fraction), cross-linker (PEGDA, 5.0% w/w relative to monomer), and radical initiator (KPS, 1.0% w/w relative to monomer) were prepared and transferred to nitrogen purged stoppered glass-reactors containing wet BNC pellicles (diameter: ca. 7 cm) with 40% of water content. After the total inclusion of the respective solution during 1 h in ice, the reaction mixtures were placed in an oil bath at 70 °C and left to react for 6 h. The nanocomposite films were then thoroughly washed with ultrapure water and oven dried at 40 °C for 12 h. Samples of cross-linked PSBMA were also prepared without BNC and all experiments were triplicated.

### 2.3. Characterization Methods

#### 2.3.1. Thickness

The thickness was measured by a hand-held digital micrometer (Mitutoyo Corporation, Tokyo, Japan) with an accuracy of 0.001 mm. All measurements were arbitrarily carried out at different spots of the films.

#### 2.3.2. Attenuated Total Reflection-Fourier Transform Infrared (ATR-FTIR)

ATR-FTIR spectra were collected in the range of 600–4000 cm^−1^ at a resolution of 4 cm^−1^ over 32 scans in a Perkin-Elmer FT-IR System Spectrum BX spectrophotometer (Perkin-Elmer, Waltham, MA, USA) equipped with a single horizontal Golden Gate ATR cell.

#### 2.3.3. Solid-State Carbon Cross-Polarization/Magic-Angle-Spinning Nuclear Magnetic Resonance (^13^C CP/MAS NMR)

^13^C CP/MAS NMR spectra were recorded on a Bruker Avance III 400 spectrometer (Bruker Corporation, Billerica, MA, USA) operating at a B0 field of 9.4 T using 12 kHz MAS with proton 90° pulse of 3 µs, time between scans of 3 s and a contact time of 2000 µs.

#### 2.3.4. Scanning Electron Microscopy (SEM)

Surface and cross-section micrographs of the films were obtained by a HR-SEM-SE SU-70 Hitachi microscope (Hitachi High-Technologies Corporation, Tokyo, Japan) operating at 4 kV. The films for the cross-sectional analysis were fractured in liquid nitrogen, then glued with conductive carbon tape on a steel plate and coated with a carbon layer before analysis.

#### 2.3.5. Thermogravimetric Analysis (TGA)

TGA was performed by a SETSYS Setaram TGA analyzer (SETARAM Instrumentation, Lyon, France) assembled with a platinum cell. Samples (ca. 5 mg) were heated from 25 to 750 °C with a constant heating rate of 10 °C min^−1^ under nitrogen atmosphere.

#### 2.3.6. Tensile Tests

Tensile tests were carried out with an Instron 5564 testing machine (Instron Corporation, Rockville, MD, USA) in the traction mode at a crosshead velocity of 10 mm min^−1^ using a 500 N static load cell. Rectangular strips (50 × 10 mm^2^) of the films were previously dried at 40 °C and equilibrated at 25 °C in a desiccator before testing.

#### 2.3.7. Ultraviolet-Visible Spectroscopy (UV–Vis)

UV-Vis spectra were acquired with a Shimadzu UV-1800 UV-Vis spectrophotometer (Shimadzu Corp., Kyoto, Japan) assembled with a quartz window plate with 10 mm diameter and recorded in steps of 1 nm in the range 200–700 nm.

#### 2.3.8. Moisture-Uptake Capacity

Dry samples (20 × 20 mm^2^) were placed in a conditioned cabinet at 98% relative humidity (RH, saturated potassium sulfate solution) at 25 °C for 48 h. After taking the samples out of the cabinet, the weight (*W*_w_) was measured and the moisture-uptake was calculated by the equation:

(1) $$\mathrm{Moisture}\;\mathrm{uptake}\;\left(\%\right)=\left(W_{w}-W_{0}\right)\times W_{0}^{-1}\times 100$$

where *W*_0_ is the initial weight of the dry film.

#### 2.3.9. Water-Uptake Capacity (and Polymer Leaching)

Dry samples (20 × 20 mm^2^) were immersed in ultrapure water at 25 °C for 48 h. After taking the samples out of the medium, the wet surfaces were dried with filter paper, and the wet weight (*W*_w_) was measured. The water-uptake was calculated by the equation:

(2) $$Water\;uptake\;\left(\%\right)=\left(W_{w}-W_{0}\right)\times W_{0}^{-1}\times 100$$

where *W*_0_ is the initial weight of the dry film. After completing the immersion tests, the samples were oven dried at 40 °C for 24 h and the dry weight measured to evaluate the cross-linked PSBMA leaching.

### 2.4. Antimicrobial Activity

The films’ antimicrobial activity was tested against *S. aureus* and *E. coli*. The bacterial pre-inoculum cultures were grown in TSB growth medium at 37 °C under shaking at 120 rpm for 24 h. Beforehand, the density of the bacterial culture was adjusted to 0.5 McFarland in PBS solution (pH 7.4) to attain a bacterial concentration of 10^8^ to 10^9^ colony forming units per mL (CFU mL^−1^). Each specimen (50 × 50 mm^2^) was placed in contact with 5 mL of bacterial suspension. A bacteria cell suspension was tested as control while BNC was tested as blank reference. All specimens were incubated under horizontal shaking at 120 rpm and 37 °C. After 24 h of contact time, 100 µL aliquots of each specimen and controls were collected and the bacteria cell concentration (CFU mL^−1^) was evaluated by plating serial dilutions on TSA medium, that were incubated at 37 °C for 24 h. The CFU were assessed on the most appropriate dilution on the agar plates. Three independent experiments were performed, and, for each, duplicates were plated. The bacterial reduction was calculated as follows: *log reduction* = *log* CFU_control_ − *log* CFU_film_.

### 2.5. Protonic Conductivity

The electrochemical impedance spectroscopy (EIS) measurements were carried out on rectangular film samples (ca. 15 × 5 mm^2^) on which two stripes of silver paste (Agar Scientific, Essex, UK) were painted at the tips of the film sample, distancing around 10 mm from each other. The samples were mounted in a tubular sample holder, using a pseudo four-electrode configuration and the electrical contact between the film and the Agilent E4980A Precision LCR meter (Santa Clara, CA, USA) was performed via platinum wires. The sample-holders were then inserted into an ACS Discovery DY110 climatic chamber (Angelantoni Test Technologies Srl, Massa Martana, Italy) in order to perform the EIS measurements in a controlled atmosphere, with variable temperature (40, 60, 80 and 94 °C) and relative humidity (60, 80 and 98%).

The impedance spectra were recorded between 20 Hz and 2 × 10^6^ Hz with 100 mV of test signal amplitude. The data was analyzed with the ZView software (Version 2.6b, Scribner Associates (Southern Pines, NC, USA)) to evaluate the Ohmic resistance (*R*) of the films. The in-plane (IP) conductivity (σ) was calculated by the equation:

(3) $$\sigma =L_{0}{\left(R\delta \omega \right)}^{-1}$$

where *L*_0_ is the distance between the two silver stripes, *δ* is the film thickness, and *w* is the film width.

### 2.6. Statistical Analysis

Analysis of variance (ANOVA) and Tukey’s test (OriginPro, version 9.0.0, OriginLab Corporation, Northampton, MA, USA) was used to determine the statistical significance established at *p* < 0.05.

## 3. Results

**Figure 1:**
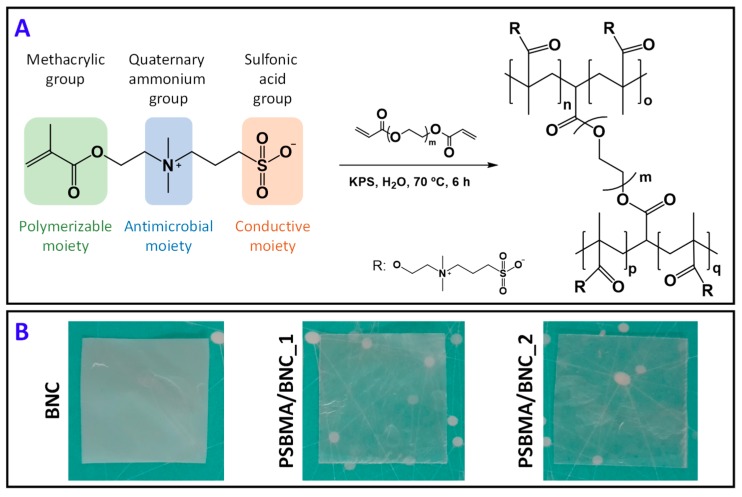
(**A**) Reactional scheme representing the radical polymerization of SBMA in the presence of poly(ethylene glycol) diacrylate (PEDGA) as cross-linker and potassium persulfate (KPS) as radical initiator, yielding cross-linked PSBMA, and (**B**) photographs of dry films of pristine BNC, and nanocomposites PSBMA/BNC_1 and PSBMA/BNC_2.

Two nanocomposite films with different contents of poly(sulfobetaine methacrylate) (PSBMA) and BNC were obtained via the one-pot in situ free radical polymerization of SBMA within the wet BNC nanofibrous network. The SBMA monomer was selected due to its functional groups, namely the methacrylic, quaternary ammonium and sulfonic acid groups, which convey the monomer with a polymerizable, antimicrobial and conductive moieties, respectively, as summarized in Figure 1A. The use of a cross-linker is intended to preserve the water-soluble zwitterionic polymer within the BNC porous structure, and poly(ethylene glycol) diacrylate (PEGDA) (Figure 1A) was carefully chosen based on earlier studies [33,34]. Figure 1B shows that the nanocomposite films are macroscopically homogeneous with no obvious agglomerates on their surfaces, which points to a good distribution of the cross-linked PSBMA zwitterionic polymer inside the BNC three-dimensional structure. The two PSBMA/BNC nanocomposites were characterized by several state-of-the-art techniques regarding structure (ATR-FTIR, ^13^C CP/MAS NMR), morphology (SEM), thermal properties (TGA), mechanical performance (tensile tests), optical properties (UV-Vis), antimicrobial activity (against *S. aureus* and *E. coli*), moisture- and water-uptake capacity, and protonic conductivity (electrochemical impedance spectroscopy), and compared with the individual components.

### 3.1. Composition and Microstructure

As previously stated, the compositions of the two nanocomposite films are distinct in terms of cross-linked PSBMA and BNC. While PSBMA/BNC_1 contains 41 ± 10 wt.% of PSBMA and 59 ± 10 wt.% of BNC, PSBMA/BNC_2 is composed of 62 ± 11 wt.% of PSBMA and 38 ± 11 wt.% of BNC (Table 1). These values correspond to nanomaterials containing 403 ± 99 mg of PSBMA per cm^3^ of film for PSBMA/BNC_1 and 661 ± 81 mg of PSBMA per cm^3^ of film for PSBMA/BNC_2. The thickness is also different among the films and augmented with the rising content of cross-linked PSBMA from 88 ± 22 µm for pristine BNC to 131 ± 24 µm for PSBMA/BNC_1 and 194 ± 55 µm for PSBMA/BNC_2 (Table 1).

**Figure 2:**
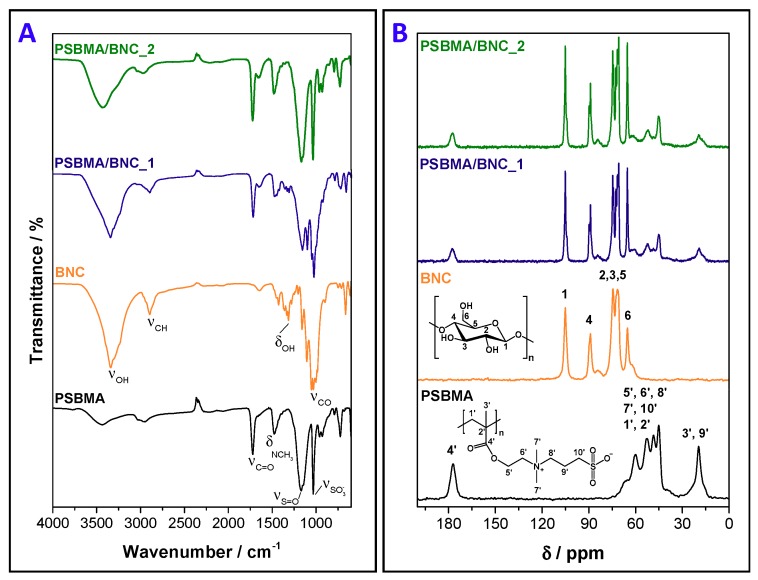
(**A**) ATR-FTIR (vibrational modes: *ν* = stretching, *δ* = bending) and (**B**) ^13^C CP/MAS NMR spectra of cross-linked PSBMA, pristine BNC, and nanocomposites PSBMA/BNC_1 and PSBMA/BNC_2.

The infrared spectra of pristine BNC, cross-linked PSBMA, and films PSBMA/BNC_1 and PSBMA/BNC_2 are displayed in Figure 2A. The ATR-FTIR spectra of the two PSBMA/BNC films present the absorption bands of cellulose at 3342 cm^−1^ (O–H stretching), 2896 cm^−1^ (C–H stretching), 1314 cm^−1^ (O–H in plane bending) and 1030 cm^−1^ (C–O stretching) [35], together with those of the cross-linked PSBMA at 1720 cm^−1^ (C=O stretching), 1478 cm^−1^ (N^+^(CH_3_)_2_ bending), 1168 cm^−1^ (S=O stretching), and 1032 cm^−1^ (${\mathrm{SO}}_{3}^{-}$ stretching) [28,36]. The existence of these absorption bands and the truancy of the one at about 1640 cm^−1^ allocated to the C=C stretching of the methacrylic group of SBMA (and cross-linker) supported the occurrence of the in situ free radical polymerization of SBMA. Furthermore, the relative intensity of the bands assigned to the cross-linked PSBMA is in accordance with the *W*_PSBMA_*/W*_total_ ratio measured for each nanocomposite (Table 1).

Figure 2B depicts the solid-state ^13^C CP/MAS NMR spectra of the films with the representative carbon resonances of cellulose at *δ* 105.1 ppm (C1), 88.9 ppm (C4), 71.6–74.5 ppm (C2,3,5) and 65.2 ppm (C6) [35], in tandem with those of the cross-linked PSBMA at *δ* 177.1 ppm (*C*=O), 60.0 ppm (O*C*H_2_*C*H_2_N*C*H_2_), 52.8 ppm (N^+^(*C*H_2_)_3_ and *C*H_2_S), 48.4 ppm (*C*H_2_ of polymer backbone), 45.2 ppm (quaternary *C* of polymer backbone) and 19.6 ppm (*C*H_3_ of polymer backbone and NCH_2_*C*H_2_CH_2_S) [27]. The fact that the resonances of the C=C double bonds of the methacrylic groups of both the SBMA (monomer) and PEDGA (cross-linker) are not visible in the respective spectra, is proof of their complete polymerization and/or elimination during the various washing steps, as formerly recognized by ATR-FTIR analysis.

**Figure 3:**
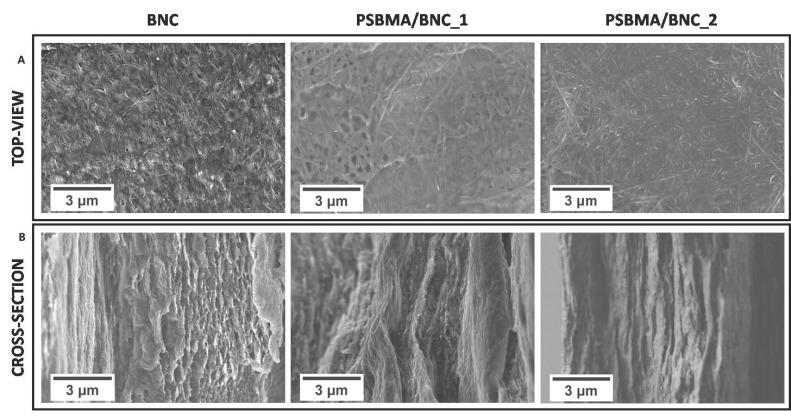
SEM images with a magnification of ×10.0 k of the (**A**) surface and (**B**) cross-section of pristine BNC and nanocomposites PSBMA/BNC_1 and PSBMA/BNC_2.

Figure 3 depicts the surface and cross-section SEM micrographs of the pristine BNC and the two nanocomposite films, viz. PSBMA/BNC_1 and PSBMA/BNC_2. A close look at the micrographs shows the three-dimensional nanofibrillar network (top-view) and lamellar microstructural features (cross-section) of the BNC morphology [37], which tend to fade away with increasing content of the zwitterion PSBMA that covers the nanofibrils and fills the lamellar spaces of the BNC network. The micrographs also show that PSBMA/BNC_2 is the film with the highest content of cross-linked PSBMA due to the fewer resemblances when compared with the characteristic morphology of BNC. Furthermore, the nanocomposite films exhibit anisotropic morphology and the cross-linked PSBMA is evenly distributed inside the BNC nanofibrous network. These trends were also observed for other nanocomposites composed of BNC and poly(4-styrene sulfonic acid) [33], BNC and poly([2–(methacryloyloxy)ethyl] trimethylammonium chloride) [37], or BNC and poly(2-methacryloyloxyethyl phosphorylcholine) [38].

### 3.2. Thermal and Mechanical Properties

**Figure 4:**
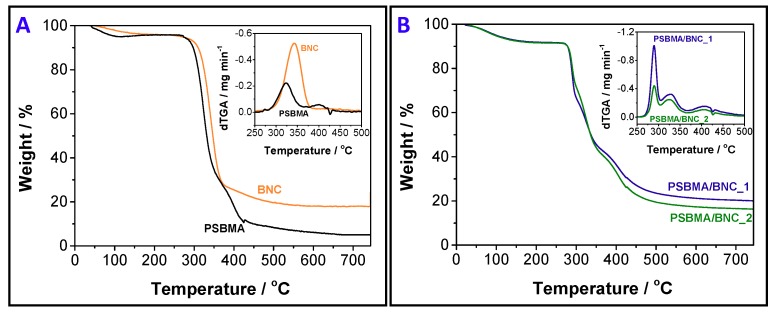
Thermograms of (**A**) cross-linked PSBMA, pristine BNC and (**B**) nanocomposites PSBMA/BNC_1 and PSBMA/BNC_2. The inset curves represent the derivative.

The thermal stability of the PSBMA/BNC nanocomposite films was evaluated by TGA and the respective data is presented in Figure 4. While the pristine BNC displays a single-step weight-loss profile with a maximum temperature of decomposition of 342 °C (final residue: ~17%) [37], the cross-linked PSBMA displays a two-step weight-loss curve with maximum temperatures of decomposition of 323 °C and 400 °C (final residue: ~5%, Figure 4A) [39]. The TGA profiles of the nanocomposite films demonstrate the combination of the two components, namely the cross-linked PSBMA and BNC, with a three-step weight-loss degradation pathway, apart from the water loss beneath 100 °C (loss of ~5%) (Figure 4B). PSBMA/BNC_1 has maximum temperatures of decomposition at 289 °C (loss of ~23%), 328 °C (loss of ~46%) and 402 °C (loss of ~64%) with a final residue of 20%, whereas for PSBMA/BNC_2 the maximum happens at 290 °C (loss of ~20%), 325 °C (loss of ~43%) and 402 °C (loss of ~68%) with a residue of 16% by the end of the analysis (750 °C). The first step is related to the pyrolysis of the cellulose skeleton [40], whose degradation decreased to a lower temperature when incorporated in the films probably because of the catalytic effect of the sulfonic acid groups, as has indeed been documented for other BNC-based materials [41] and sulfonated-BNC [42]. The second and third weight-loss steps are allocated to the thermal degradation of the zwitterionic polymer backbone and pendant groups, namely the ester, quaternary ammonium, and sulfonate groups [39]. Thus, Figure 4B clearly demonstrates that the nanocomposite films are thermally stable up to 265 °C with a weight-loss of ~8.5% that includes the 5% dehydration below 100 °C.

The mechanical properties of the PSBMA/BNC films were studied via tensile tests and the data from the stress-strain curves are summarized in Table 2. While the pristine BNC film exhibits good mechanical properties with Young’s modulus of 17.8 ± 2.3 GPa, tensile strength of 207 ± 20 MPa and elongation at break of 1.5 ± 0.7%, the lack of data regarding the cross-linked PSBMA is explained by its film-forming inaptitude. Globally, the mechanical performance of the nanocomposite films increased with the growing content of cellulose, as previously described for other BNC-based nanocomposites [38,43,44]. The augmentation of the BNC content from 38 wt.% to 59 wt.% caused an increase of the Young’s modulus and tensile strength from 3.1 ± 0.4 GPa and 28 ± 4 MPa (PSBMA/BNC_2) to 4.6 ± 0.5 GPa and 43 ± 7 MPa (PSBMA/BNC_1), respectively. For the elongation at break, there is no significant differences and both nanocomposite films have lower values just like the pristine BNC film (Table 2). The comparison of this data with the figures reported by Padrão et al. [23] regarding BNC/lactoferrin edible films points to an analogous trend regarding the Young’s modulus, tensile strength and elongation at break. Still, the present PSBMA/BNC nanocomposite films exhibit superior mechanical properties.

The conjunction of the results of the thermal and mechanical properties show that these films can endure (i) sterilization under typical procedures at ca. 150 °C, such as autoclaving, which is every so often a prerequisite for food-based applications, and (ii) the mechanical stress during handling, storage and transport as food packaging materials.

### 3.3. Optical Properties

**Figure 5:**
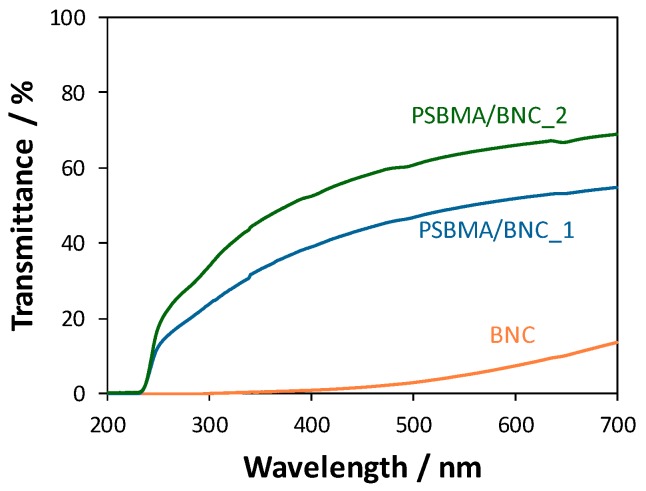
UV-vis transmission spectra of pristine BNC and nanocomposites PSBMA/BNC_1 and PSBMA/BNC_2.

The UV-vis data in the range 200–700 nm for pristine BNC and both PSBMA/BNC nanocomposites is displayed in Figure 5. The transmittance of the BNC film (thickness: 88 ± 22 µm, Table 1) only begins to rise in the visible range (400–700 nm) and reaches a maximum of ca. 13.5% at 700 nm, which shows its low transparency in accordance with the BNC white color (Figure 1B). Additionally, BNC has UV-absorbing properties in the UVA (320–400 nm, long-wavelength radiation), UVB (280–320 nm, short-wavelength radiation) and UVC (100–280 nm, short-wavelength radiation) regions with transmittance values below 1%, in accord with data described in literature [45,46].

After the inclusion of PSBMA into the BNC 3D network, the films significantly became more transparent as demonstrated in Figure 1B (macroscopic appearance) and corroborated by the transmittance values of 39–55% for PSBMA/BNC_1 and 52–69% for PSBMA/BNC_2 in the visible range (Figure 5). In the ultraviolet region (200–400 nm), the transmittance continued under 15% until 260 nm for PSBMA/BNC_1 and 250 nm for PSBMA/BNC_2, and then gradually increased to 39% and 52% at 400 nm for PSBMA/BNC_1 and PSBMA/BNC_2, respectively. Moreover, PSBMA/BNC_2 exhibits bigger transmittance values and concurrently inferior absorbance values, which represents a transmittance increase with higher content of cross-linked PSBMA (Figure 5). A parallel behavior was described for other BNC-based nanocomposites comprising for instance poly([2–(methacryloyloxy)ethyl] trimethylammonium chloride) [37], poly(2-methacryloyloxyethyl phosphorylcholine) [38] and poly(ε-caprolactone) [47].

The barrier properties against UV-light are important in the context of the prevention of photo-oxidation of light-sensitive food products like for example ham and drinks. Therefore, the UV-blocking properties of the PSBMA/BNC films might contribute to extend the shelf-life of foodstuffs since these films can act as UV absorbers, which are indeed secondary (or preventive) antioxidants for application in antioxidant active packaging systems [19].

### 3.4. Antimicrobial Activity

For active food packaging, the utilization of antimicrobial agents that can inhibit the growth of pathogenic and/or spoilage microorganisms will contribute to limit food spoilage [19]. The zwitterion PSBMA cross-linked polymer contains a quaternary amine group known for its antimicrobial activity [26,27], and thus the PSBMA/BNC nanocomposite films are expected to exhibit antimicrobial activity. The validation of this conjecture was performed by determining the growth inhibition of gram-positive (*S. aureus*) and gram-negative (*E. coli*) bacteria, which are two key pathogenic microorganisms responsible for food spoilage and foodborne illness outbreaks [48,49,50].

**Figure 6:**
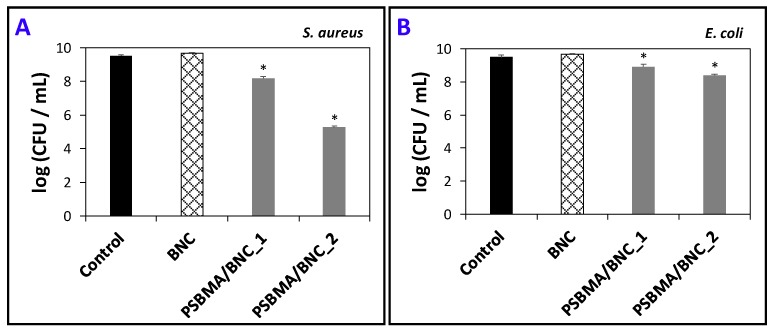
Effect of pristine BNC, PSBMA/BNC_1 and PSBMA/BNC_2 on the bacterial concentration of (**A**) *S. aureus* and (**B**) *E. coli* after 24 h of exposure; error bars represent the standard deviation; the asterisk (*) denotes statistically significant differences to the control treatment (*p* < 0.05).

Figure 6 summarizes the antimicrobial activity of both PSBMA/BNC nanocomposites and of the pristine BNC film. The pristine BNC film, together with the experimental control where the two bacteria were inoculated in culture media without any films, did not disturb the viability of both *S. aureus* and *E. coli*. This was anticipated because according to literature the exopolysaccharide BNC does not inhibit the growth of *S. aureus*, *E. coli* [23,38,51], *Candida albicans* [37], *Pseudomonas aeruginosa* and *Bacillus subtilis* [51]; actually, BNC can be the substrate for microbial cell culture [52].

The PSBMA/BNC_1 nanocomposite presents low antimicrobial activity towards both *S. aureus* and *E. coli* with 1.3–and 0.6–log CFU mL^−1^ reduction, respectively. The PSBMA/BNC_2 also exhibits low antimicrobial activity towards *E. coli* with 1.1–log CFU mL^−1^ reduction but has high bactericidal activity against *S. aureus* with 4.3–log CFU mL^−1^ reduction (Figure 6). This is a clear indication that the incorporation of cross-linked PSBMA into the BNC 3D network is responsible for the demise of both gram-positive and gram-negative bacteria. According to the literature, PSBMA has caused the reduction of both *S. aureus* and *E. coli* after being grafted to a poly(vinyl alcohol)-formaldehyde (PVF) sponge, particularly in the case of the gram-negative bacterium for which the pure PVF has absolutely no antimicrobial activity [27]. In this sense, the aptitude of the PSBMA/BNC nanocomposite films to inhibit the growth of both *S. aureus* and *E. coli* bacteria validates their potential applicability for antimicrobial food packaging.

### 3.5. Moisture- and Water-Uptake Capacity

SBMA is a hygroscopic monomer with the ability to absorb environmental humidity. Thus, the moisture-uptake capacity of the PSBMA/BNC films was assessed to investigate their ability to absorb moisture that so deeply affects humidity-sensitive foodstuffs. Therefore, the films were placed in a chamber with controlled humidity (98% RH) at RT for 48 h. According to Table 3, the cross-linked PSBMA polymer is hygroscopic just like the respective monomer with a moisture-uptake capacity of 65 ± 1%, and its inclusion into the BNC porous structure yielded nanocomposite films capable of absorbing 27 ± 1% (0.27 ± 0.01 g of moisture *per* g of film) for PSBMA/BNC_1 and 36 ± 2% (0.36 ± 0.02 g of moisture *per* g of film) for PSBMA/BNC_2.

The next step was to evaluate the water-uptake capacity by immersing the films in water at 25 °C for 48 h. As listed in Table 3, the water-uptake results show that this parameter increased with the rising content of cross-linked PSBMA. While the pristine BNC film can absorb 79 ± 4% of water, the two nanocomposite films have a water-uptake capacity of 450 ± 14% for PSBMA/BNC_1 and 559 ± 15% for PSBMA/BNC_2. These values demonstrate that the incorporation of 41 and 62 wt.% of cross-linked PSBMA into the BNC network promoted an augment of 5.6 and 7.1 times, respectively, of the water-uptake capacity of the pristine BNC film. In fact, this behavior was predictable given the super-hydrophilic nature of the zwitterionic polymer as reported by Lalani and Liu [26]. Furthermore, the PSBMA/BNC nanocomposite films exhibit water-uptake values relatively comparable to those obtained for pure electrospun PSBMA membranes (354%), but much higher than the value of about 81% attained for PSBMA hydrogels [26].

Noteworthy is the fact that after 48 h of immersion in ultrapure water, the two nanocomposite films were dried and the final weights pointed to polymer losses between 1–3%, which underlines the minimal leaching of the cross-linked PSBMA out the BNC three-dimensional network. In addition, the dried films were tested again for water uptake, and the values remained fairly the same, thus confirming the reversibility of the water-uptake ability and the possible reusability of the PSBMA/BNC films.

The fact that the PSBMA/BNC nanocomposites absorb moisture is pertinent in the framework of active food packaging since these films can act as moisture scavengers to extent food shelf-life by preserving the moisture content and decreasing the condensation of moisture in the food package [22]. Furthermore, their ability to absorb substantial amounts of water is also pertinent in the event of moisture condensation in the food package and liquid release from fresh products like fish, meat and fruit/vegetables [22]. Thus, these PSBMA/BNC films can contribute to reduce moisture content and water activity in moisture-sensitive food, and consequently the food will be less prone to microbial spoilage, which on the other hand increases shelf-life.

### 3.6. Protonic Conductivity (Proton Motion)

PSBMA/BNC nanocomposite films have sulfonic acid groups in their structure (Figure 1A) which are protogenic moieties known for enabling proton motion [33]. This property is important in the context of intelligent food packaging (i.e., indicators, data carriers and sensors that check the state of packaged food products [20]) particularly when addressing protonic-conduction humidity sensors to monitor humidity levels during transportation and storage of food products that cannot tolerate high environmental humidity. In fact, poor manipulation of sealed packaged food leading to package rupture and condensation caused by continued exposure to extreme temperature variations, are two of the major causes of humidity fluctuations in packaged foodstuffs [53]. The class of relative humidity sensors are the simplest, cheapest and most used commercial sensors where RH is the unit of measurement, as opposed to absolute humidity. These RH sensors can utilize the ionic type humidity-sensing operating principle to monitor changes in ionic conductivity upon exposure to different humidity levels, as discussed in the review by Farahani et al. [54]. Thus, the study of the protonic conductivity of these zwitterionic films will provide information about proton motion to evaluate their possible applicability for protonic-conduction humidity sensors.

**Figure 7:**
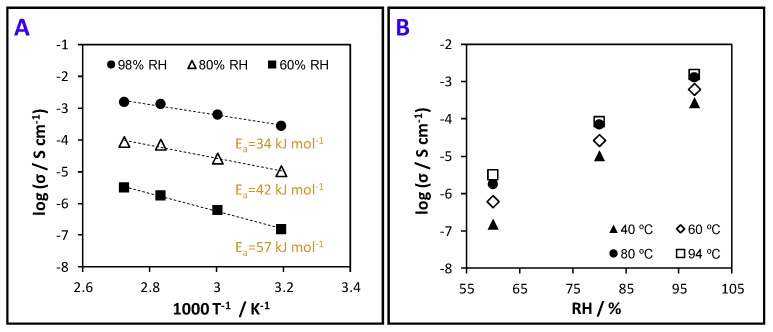
(**A**) Arrhenius-type plot of the IP conductivity of the film PSBMA/BNC_2 at different RH; the straight lines are linear fits to the Arrhenius model, and (**B**) conductivity logarithm as a function of RH under variable temperature.

Given that PSBMA/BNC_2 exhibits the highest content of sulfonic acid groups, this nanocomposite film was selected for assessing the protonic conductivity by electrochemical impedance spectroscopy. Hence, Figure 7A represents the Arrhenius-type plots for the protonic conductivity of PSBMA/BNC_2 film measured at distinct temperatures and Figure 7B represents the conductivity as a function of relative humidity. The latter parameter is the one accountable for the larger differences with the protonic conductivity increasing three orders of magnitude from 1.5 × 10^−4^ mS cm^−1^ at 60% RH to 2.8 × 10^−1^ mS cm^−1^ at nearly saturated conditions (98%) and 40 °C (Figure 7B). Contrary to the RH, the temperature increase leads to a more modest augment of the protonic conductivity by one order of magnitude, from 2.8 × 10^−1^ mS cm^−1^ at 40 °C to 1.5 mS cm^−1^ at 94 °C at a RH of 98% (Figure 7A). Furthermore, the conductivity data shown in Figure 7A can be fitted by the Arrhenius equation:

(4) $$\sigma =\sigma_{0}e^{-E_{a}{\left(RT\right)}^{-1}}$$

where *σ*_0_ is a pre-exponential term, *E*_a_ is the activation energy for proton transport, *R* is the gas constant and *T* is the absolute temperature. For the PSBMA/BNC_2 film the estimated *E*_a_ values decreased with increasing humidity in the range of 34–57 kJ mol^−1^, which is naturally linked with the structural diffusion of protons as the prime mechanism of transport [55]. These figures, together with the reported low conductivity of BNC (ca. 60 × 10^−3^ mS cm^−1^ at 94 °C, 98% RH) [33], decoded the sulfonic acid groups as the culprits for the protonic conductivity of the nanocomposite film, alongside its high water-uptake capacity (559 ± 15%, Table 3).

Despite the lower protonic conductivity of the PSBMA/BNC film in comparison with, for instance, the benchmark Nafion™ ionomer (conductivity of ca. 100 mS cm^−1^ (94 °C, 98% RH) and water-uptake of 54% [56]) that was recently used as a humidity sensor [57], the attained values are in tune with the zwitterionic nature of the cross-linked PSBMA. For example, Guo et al. [58] stated that the anchoring of SBMA to ZIF-8 (zeolitic imidazolate framework) originated a composite membrane with a protonic conductivity of 17.2 mS cm^−1^ at 65 °C. Thus, the protonic conductivity of the PSBMA/BNC_2 film envisages their potential use as humidity sensors based on protonic conductivity as a function of water content, *viz*. conductimetric humidity sensors [54].

The zwitterion PSBMA/BNC nanocomposites benefit from a combination of properties, namely UV-light protection, moisture- and water-uptake capacity, antimicrobial activity, and protonic conductivity, that can be fine-tuned by altering the amount of the individual components. This catalogue of features is vital in the field of active food packaging [19], where the films should exhibit barrier properties against UV light to avert photo-oxidation of light-sensitive food products, as well as moisture scavenging to reduce moisture content and water activity in moisture-sensitive food, and antimicrobial activity to avoid the growth of pathogenic and/or spoilage microorganisms [19]. Additionally, the protonic conductivity of the films can be a major asset for intelligent food packaging, where proton motion plays an important role in protonic-conduction humidity sensors [54] that detect humidity levels in foodstuffs (e.g., dry foods, dairy and meat products) that cannot tolerate high environmental humidity [53]. Altogether, this will contribute to extending food product quality or shelf-life and concomitantly to reducing spoilage, food waste and foodborne illness outbreaks [19].

## 4. Conclusions

Zwitterion films composed of poly(sulfobetaine methacrylate) and bacterial nanocellulose were successfully developed through the one-pot polymerization of sulfobetaine methacrylate inside the BNC nanofibrous network. The films are optically transparent and present thermal stability up to 265 °C in nitrogen atmosphere, high mechanical properties (Young’s modulus ≥ 3.1 GPa), and water-uptake capacity (450–559%). Moreover, the films display barrier properties against UV radiation. The zwitterion film with 62 wt.% of cross-linked PSBMA disclosed a maximum proton conductivity of ca. 1.5 mS cm^−1^ (at 94 °C and 98% RH), bactericidal effect against *S. aureus* (4.3–log CFU mL^−1^ reduction) and *E. coli* (1.1–log CFU mL^−1^ reduction). The deluge of findings in the present study about these zwitterion nanocomposite films reflect the possibility for application as active and intelligent food packaging materials, since these films can shield the food from the adverse effects of UV radiation, hinder the growth of foodborne pathogens, absorb moisture and water, and act as protonic-conduction humidity sensors to monitor humidity levels in foodstuff.

## Figures and Tables

**Table 1:** List of the studied films with their weight compositions and thicknesses.

Samples	Composition ^a^			Thickness/µm
	*W*_BNC_/*W*_total_	*W*_PSBMA_/*W*_total_	*W*_PSBMA_/*V*_total_ (mg cm^–3^) ^b^	
**BNC**	1.0	–	–	88 ± 22
PSBMA/BNC_1	0.59 ± 0.10	0.41 ± 0.10	403 ± 99	131 ± 24
PSBMA/BNC_2	0.38 ± 0.11	0.62 ± 0.11	661 ± 81	194 ± 55

^a^ Composition was calculated by taking into account the weight of the film (*W*_total_), BNC (*W*_BNC_) and PSBMA cross-linked polymer (*W*_PSBMA_ = *W*_total_ − *W*_BNC_); ^b^ Ratio between the mass of the cross-linked PSBMA (*W*_PSBMA_) and the volume of the film (*V*_total_); all values are the mean of triplicates with the respective standard deviations.

**Table 2:** Young’s modulus, tensile strength and elongation at break of the pristine BNC and the PSBMA/BNC nanocomposite films; all values are the mean of five replicates with the respective standard deviations; the asterisk (*) denotes statistically significant differences with respect to the pristine BNC (*p* < 0.05).

Film.	Young’s Modulus/GPa	Tensile Strength/MPa	Elongation at Break/%
BNC	17.8 ± 2.3	207 ± 20	1.5 ± 0.7
PSBMA/BNC_1	4.6 ± 0.5 *	43 ± 7 *	0.7 ± 0.2
PSBMA/BNC_2	3.1 ± 0.4 *	28 ± 4 *	0.8 ± 0.4

**Table 3:** Moisture- and water-uptake capacity of the PSBMA/BNC nanocomposites and the corresponding individual components; all values are the means of three replicates with the respective standard deviations.

Samples	Moisture-Uptake/%	Water-Uptake/%
BNC	14 ± 2	79 ± 4
PSBMA	65 ± 1	–
PSBMA/BNC_1	27 ± 1	450 ± 14
PSBMA/BNC_2	36 ± 2	559 ± 15
